# Multivariate Regression in Conjunction with GA-BP for Optimization of Data Processing of Trace NO Gas Flow in Active Pumping Electronic Nose

**DOI:** 10.3390/s23031524

**Published:** 2023-01-30

**Authors:** Pengjiao Sun, Yunbo Shi, Yeping Shi

**Affiliations:** 1 The Higher Educational Key Laboratory for Measuring & Control Technology and Instrumentation of Heilongjiang Province, Harbin University of Science and Technology, Harbin 150080, China; 2 Electronics and Communication Engineering School, Jilin Technology College of Electronic Information, Jilin 132021, China; 3 Heilongjiang Province Key Laboratory of Laser Spectroscopy Technology and Application, Harbin University of Science and Technology, Harbin 150080, China; 4 National Experimental Teaching Demonstration Center for Measurement and Control Technology and Instrumentation, Harbin University of Science and Technology, Harbin 150080, China

**Keywords:** active pumping, electronic nose system, GA-BP, multiple regression, trace-gas detection

## Abstract

Exhaled nitric oxide trace gas at the ppb level is a biomarker of human airway inflammation. To detect this, we developed a method for the collection of active pumping electronic nose bionic chamber gas. An optimization algorithm based on multivariate regression (MR) and genetic algorithm–back propagation (GA-BP) was proposed to improve the accuracy of trace-level gas detection. An electronic nose was used to detect NO gas at the ppb level by substituting breathing gas with a sample gas. The impact of the pump suction flow capacity variation on the response of the electronic nose system was determined using an ANOVA. Further, the optimization algorithm based on MR and GA-BP was studied for flow correction. The results of this study demonstrate an increase in the detection accuracy of the system by more than twofold, from 17.40%FS before correction to 6.86%FS after correction. The findings of this research lay the technical groundwork for the practical application of electronic nose systems in the daily monitoring of FeNO.

## 1. Introduction

An electronic nose is an analytical device used for the rapid detection and identification of gases. Due to its favorable properties, this device is used in widespread applications in diverse industries, such as agriculture, food, and environmental monitoring industries [1,2,3,4]. Owing to the rising demand for non-invasive methods in daily health-monitoring devices, there has been a recent surge in studies on electronic nose technology in the medical field [5,6]. However, the concentrations of a significant number of human biomarkers are extremely low, and they are in the order of parts per billion by volume (parts per billion, ppb) [7,8]. This has facilitated the need to enhance the detection capabilities and data processing methods of trace-gas electronic nose technology [9]. Human fractional exhaled nitric oxide (FeNO) is a biomarker of airway inflammation [10,11]. According to the *Guidelines for the Clinical Use of eNO* [12,13], which was jointly developed by the American Thoracic Society and the European Respiratory Society, the concentration of FeNO in normal adults is <25 ppb (<20 ppb for pediatrics), while that in patients with asthma is >50 ppb (>35 ppb for pediatrics). Nevertheless, some people with asthma do not perceive their symptoms sufficiently well [14,15] and, thus, do not realize that their condition is deteriorating. By monitoring FeNO, patients can compare their daily symptoms with the monitoring results, and, subsequently, this can help them to manage and avoid the worsening of their asthma in a timely manner [16]. According to the type of conversion signal, common NO sensors can be categorized as either electrochemical or optical sensors [17,18]. The disadvantages of optical sensors include a poor long-term stability, reliability, and consistency; a short service life; and a relatively high price [19,20]. The advantages of electrochemical sensors include a high sensitivity, a low error rate, simple operation, and rapid detection [21]. Electrochemical-sensor-based electronic noses possess the ability to detect small biomarkers, including those that have low ppb levels [22,23]. These devices produce an electrical signal that is proportional to the gas concentration based on a reaction with the measured gas. The NIOX MINO^®^ (Aerocrine AB) detection system, which is frequently adopted in clinical practice, functions according to electrochemical principles [24,25,26]. The superiority of electronic noses over other common exhaled-gas detection techniques, such as gas chromatography (GC), mass spectrometry (MS), optical-based breath detection techniques, and exhaled-gas condensate detection, is demonstrated by various properties, such as their simplicity of operation, rapid response time, low cost, and easy miniaturization [27,28,29,30,31].

Gas sampling approaches, such as the headspace, pump, and enrichment sampling approaches, are important elements of electronic nose detection systems. Pump sampling integrates a pneumatic pump to pump gas into the measurement area in order to ensure that it can quickly flow through the sensor surface. The advantages of pump sampling include a rapid detection speed, the abilities to sample gas in a circular fashion and to stabilize the airflow on the sensor surface, and good resistance to environmental pressure. In any system, it is important to detect ppb-level trace gases and to subsequently stabilize the gas flow quickly. In an electronic nose detection system, the flow rate of the gas that is to be measured has a direct impact on the sensor response [32]. Sedlák et al. [33] improved gas detection by adjusting the flow rate of the gas to be measured around the current sensor. They demonstrated that, for a constant concentration of detected gas, the spectral density of the current fluctuations changes significantly in terms of the level and shape as the flow rate increases. In addition, evaluating these fluctuations and the direct current components helped to compensate for the adverse impact of the flow rate on the sensor responses. Tan et al. [34] found that the responses of thin-film gas sensors were significantly dependent on the velocity of the gas that was measured mainly because it flowed over the sensor surface. The impact of gas velocity on the sensor response varied for several gases that were measured. Liu et al. [35] studied the impact of gas flow on the sensitivity of a membrane-covered oxygen sensor. The significance of the effect of the gas flow rate on the sensor response was confirmed, and the relationship between the sensor response and the gas flow rate was explored. Gębicki et al. [36] analyzed the impact of the volumetric flow rate on the signal and response time of a Nafion membrane sulfur dioxide gas sensor. The sensor signals and response times were analyzed for flow rates in the range of 0–100 cm^3^/min. However, these studies did not integrate machine learning algorithms to correct the pump flow rate and sensor response.

To improve the detection accuracy of trace gases according to the concentration interval of FeNO, this study simulated the NO exhaled by the human body based on a standard sample gas, placed a NO electrochemical sensor in a bionic chamber, and adopted a pumping gas sampling method to establish a bionic-chamber-based electronic nose detection system [37]. The impact of the pump suction flow on the system’s detection accuracy was determined using ANOVA, and a multivariate regression (MR) and genetic algorithm–back propagation (GA-BP) correction method was proposed to improve the detection accuracy of the system. This provides a basis for the study of health-monitoring equipment that can take daily measurements of FeNO and also serves as a method for optimizing the processing of trace-gas detection data using electronic nose technology.

## 2. Methods

### 2.1. System Design

**Figure 1:**
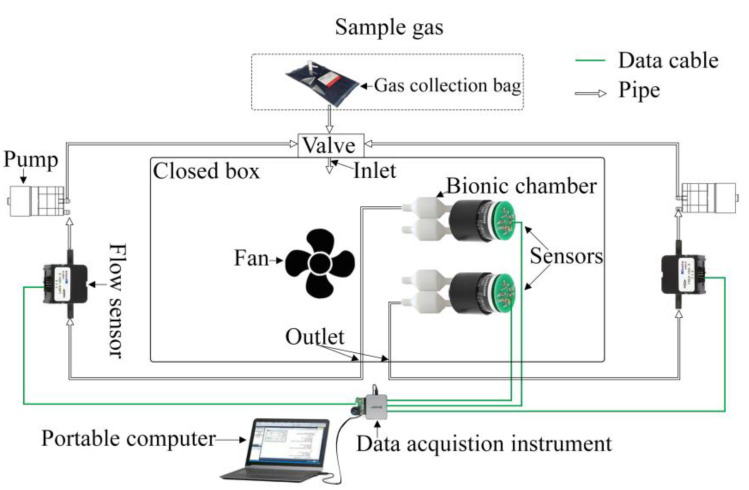
Schematic of the experimental electronic nose detection system.

The electronic nose detection system primarily consists of a closed box, a NO sensor, a bionic chamber, an air pump, a fan, a flow sensor, a data acquisition card, and a computer. A schematic of the device is illustrated in Figure 1. The range of trace gases to be measured at the ppb level was determined based on the NO concentration interval that is exhaled by the human body, and a standard gas was used instead. To ensure that a constant gas concentration was maintained in the closed box and that there was sufficient contact between the trace gas and the NO sensor, the closed box was designed by circulating the airflow. The bionic-chamber-equipped sensor was placed at the beginning of the circulating airflow. The air pump caused the measured gas to produce a circulating airflow. The size and shape of the outlet port were the same as those of the inlet port, and the gas was extracted from the closed box via the outlet port and returned via the inlet port.

### 2.2. Modeling and Simulation

AIRPAK software was used to model and simulate the structure of the closed box and the flow rate of the sampling gas. The gas within the closed box simulated human exhalation. The locations of the inlet and outlet of the closed box were determined according to the collected molar quantity of the gas that was to be measured and the fluid characteristics of the gas molecules. The difference in the system response due to different pump suction flow capacities was investigated. The research was predicated on the predetermined length, width, and height of the closed box, and it aimed to collect trace gases offline. The simulations were conducted in an environment with a constant temperature and humidity.

The suction flow of the pump in the bionic chamber of the electronic nose was modeled and simulated using ANSYS software. Different pump suction flow capacities were applied at the entrance of the bionic chamber. The flow velocity and eddy viscosity at the sensor position were numerically simulated to study the impact of the pump suction flow capacity on the airflow in the bionic chamber of the electronic nose system.

### 2.3. Correlation Discrimination between Pump Suction Flow and Sensor Response

The impact of the pump suction flow of the sampling gas on the sensor response was observed using a two-factor ANOVA. The pump suction flow and the sampling gas concentration were used as the factors, and the output voltage of the sensor was used as the dependent variable. The moving-average method was used for the data preprocessing of the NO gas sensor and to obtain the smooth response voltages. Further, the loess smoothing filter method was used for the data preprocessing of the flow sensor response. Five eigenvalues were extracted from the preprocessed data—the maximum, mean, median, first quartile (Q1), and third quartile (Q3). The calculations were performed based on the equation presented below, and the impact of the sampling gas pump suction flow capacity on the sensor response was subsequently analyzed using an F-test.

(1) $$SS_{T}=SS_{A}+SS_{B}+SS_{AB}+SS_{E},$$

where is the total deviation of the response value; $SS_{A}$ and $SS_{B}$ are the deviation caused by the independent action of the pump suction flow capacity and the concentration of the sampling gas, respectively; $SS_{AB}$ is the deviation caused by the interaction between the pump suction flow capacity and concentration; and $SS_{E}$ is the error caused by random factors.

(2) $$SS_{A}=st\sum_{i=1}^{r}{\left(\bar{X_{i}}-\bar{X}\right)}^{2},$$

(3) $$SS_{B}=rt\sum_{j=1}^{s}{\left(\bar{X_{j}}-\bar{X}\right)}^{2},$$

(4) $$SS_{AB}=t\sum_{i=1}^{r}\sum_{j=1}^{s}{\left(\bar{X_{ij}}-\bar{X_{i}}-\bar{X_{j}}+\bar{X}\right)}^{2},$$

(5) $$SS_{E}=\sum_{k=1}^{t}\sum_{i=1}^{r}\sum_{j=1}^{s}{\left(X_{ijk}-\bar{X_{i}}{}_{j}\right)}^{2},$$

where r is the number of gas pump suction flow capacity values for factor A, s is the number of sampling gas concentration values for factor B, and t is the number of interactions between factors A and B.

(6) $$F_{A}=\frac{SS_{A}}{df_{A}}/\frac{SS_{E}}{df_{E}}$$

(7) $$F_{B}=\frac{SS_{B}}{df_{B}}/\frac{SS_{E}}{df_{E}},$$

(8) $$F_{AB}=\frac{SS_{AB}}{df_{AB}}/\frac{SS_{E}}{df_{E}},$$

where df is the degree of freedom.

### 2.4. Flow Correction Method

**Figure 2:**
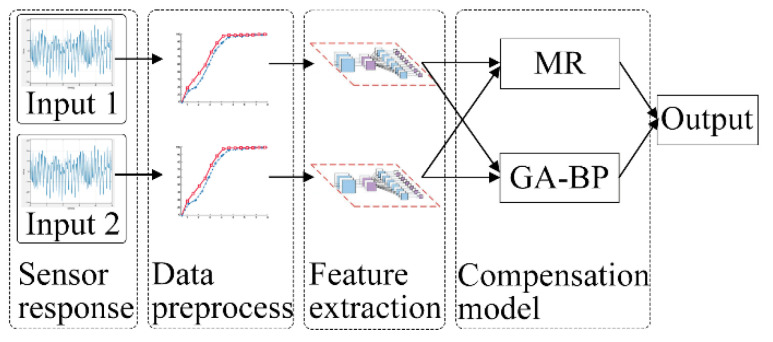
Modified flow model. Input 1 and Input 2 are response voltages of the NO gas sensor and flow sensor, respectively.

The modified flow model consisted of two inputs and one output, as illustrated in Figure 2. The two inputs were the responses of the pump flow sensor and the NO sensor, and the output was the concentration of the standard gas. All experimental gases were derived from the same standard gas.

(1) Multiple regression algorithm

We assume that the dependent variable y is affected by the k independent variables $x_{1},\;x_{2},\;\ldots ,\;x_{k}$; its n groups of observed values are $\left(y_{a},\;x_{1a},\;\ldots ,\;x_{ka}\right)$, where a=1, 2,… , n. Then, the structural form of the multiple regression equation is

(9) $$y_{a}=\beta_{0}+\beta_{1}x_{1a}+\beta_{2}x_{2a}+\ldots +\beta_{k}x_{ka}+\varepsilon_{a},$$

where $\beta_{0},\;\beta_{1},\;\ldots ,\;\beta_{k}$ are the parameters to be measured, and $\varepsilon_{a}$ is a random variable. If $b_{0},\;b_{1},\;\ldots ,\;b_{k}$ are the respective fitting values of $\beta_{0},\;\beta_{1},\;\ldots ,\;\beta_{k}$, then

(10) $$\hat{y_{a}}=b_{0}+b_{1}x_{1}+b_{2}x_{2}+\ldots +b_{k}x_{k},$$

where $b_{0}$ is a constant, and $b_{0},\;b_{1},\;\ldots ,\;b_{k}$ are the partial regression coefficients.

According to the principle of least squares,

(11) $$Q=\sum_{a=1}^{n}(y_{a}-\hat{y_{a}}{)}^{2}=\sum_{a=1}^{n}[y_{a}-(b_{0}+b_{1}x_{1}+b_{2}x_{2}\ldots +b_{k}x_{k}){]}^{2}\to \min$$

The linear fitting model and the quadratic fitting model were chosen, and the response voltages of the NO sensor and the pump suction flow capacity were used for the fitting calculation. The coefficient of the regression equation was obtained based on a comparison of the *t*-test and the F-test, and the flow correction equation, which was obtained using the multiple regression algorithm, was determined.

(2) GA-based BP neural network algorithm

The modified model of the GA-BP neural network possessed two inputs and one output, and the hidden layer contained two neurons. The formula for the neuron is

(12) $$y=f(\sum_{i=1}^{n}\omega_{i}x_{i}-\theta )\;i,j=1,2,\ldots ,n,$$

where $x_{i}$ is the input training data set, $\omega_{i}$ is the weight, y is the output of neurons, θ is the threshold, and f is the activation function.

We then input the training set $D\;=\;{\left\{\left(x_{k},y_{k}\right)\right\}}_{k=1}^{m}$. For data ${\left(x_{k},\;y_{k}\right)}_{j}$, we assume that the neural network’s output is $\hat{y_{j}^{k}}$ and that the mean square error of the network ${\left(x_{k},\;y_{k}\right)}_{j}$ is $E_{k}$.

(13) $$\begin{array}{cc} E_{k}=\frac{1}{2}\sum_{j=1}^{l}(\hat{y_{j}^{k}}-y_{j}^{k}{)}^{{}^{2}} & j=1,2,\ldots n \end{array},$$

(14) $$E=\frac{1}{m}\sum_{k=1}^{m}E_{k}\to \min,$$

The BP algorithm is based on a gradient descent strategy, which adjusts the parameters in the negative gradient direction of the target, minimizes the accumulated error E in the training set, and then determines the multilayer feedforward neural network model according to the weight and threshold.

The choices of network structure, initial weights, and thresholds have a significant impact on the network training. The initial weights and thresholds in the BP neural network algorithm are randomly generated, and they may fall into local minima during the training process as the size of the data set increases. With regard to the aforementioned characteristics, this study optimizes a neural network using GA sampling.

A GA is an evolutionary algorithm that encodes problem parameters into chromosomes and iteratively performs various operations, such as selection, crossover, and mutation, to screen individuals and to subsequently identify those with high fitness values. A BP neural network algorithm that is based on a GA, with the pump suction flow capacity and the sensor response of the sampling gas as the input training data and the content of the sampling gas as the output training data, helps to obtain the optimal initial weights and thresholds. Further, it can determine the GA-BP training model and calculate the prediction results accordingly.

(3) Optimization algorithm based on MR and GA-BP

**Figure 3:**
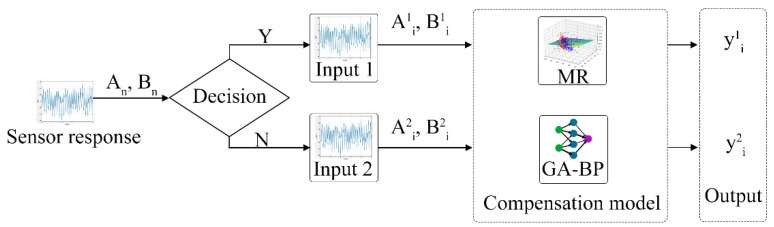
Flow compensation model of pumping electronic nose, where A^m^_n_ is NO sensor response data; B^m^_n_ is flow sensor collecting data; and m and n are group number and serial number, respectively.

The MR algorithm used in this study is based on the linear regression approach. In contrast, the GA-BP algorithm uses nonlinear regression, and both approaches have their respective advantages. The calculation concept based on the MR and GA-BP optimization algorithm combines the predictive data advantages of both approaches, as illustrated in Figure 3. Initially, two trained computational models are obtained using these algorithms. Subsequently, the data in the data set are automatically selected for the different training models based on the prediction error discrimination in order to obtain optimally corrected data.

## 3. Experiment and Simulation Process

### 3.1. Experimental Equipment

**Figure 4:**
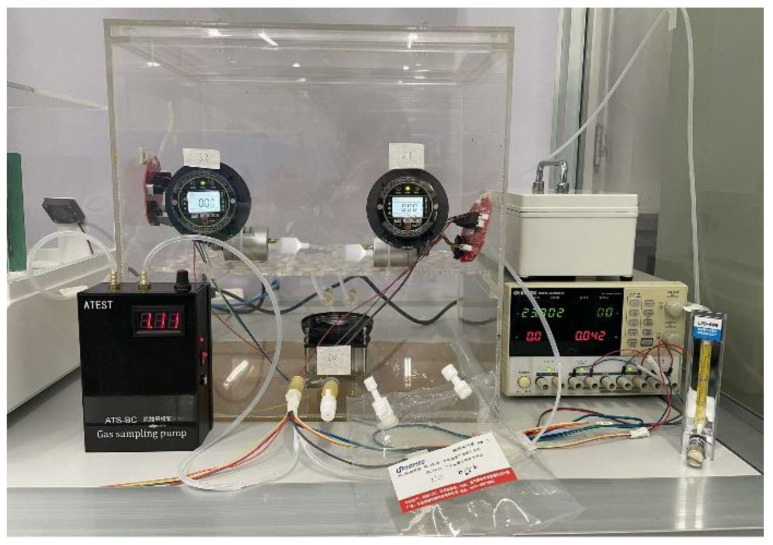
Experimental setup of electronic nose detection.

**Figure 5:**
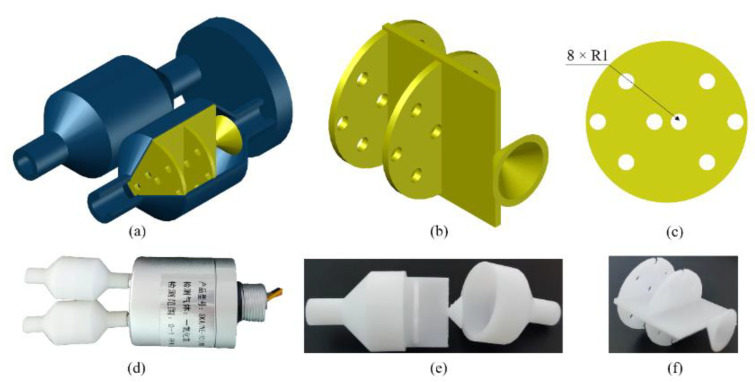
Electronic nose bionic nasal chamber: (**a**) three-dimensional model, (**b**) bionic partition in chamber, (**c**) single sieve plate in bionic partition, (**d**) physical picture of electronic nose, (**e**) physical picture of single bionic nasal chamber, and (**f**) physical picture of bionic baffle.

The experimental platform of the electronic nose system is shown in Figure 4. The volume of the closed box was 48 L (0.3 × 0.4 × 0.4 m). The Winsen FR03H model was used as the gas flow sensor. The electronic nose bionic chamber that mimicked canine nasal sieve turbinates was fabricated using Zrapid SLA880, as shown in Figure 5. The diameter and length of the chamber were 20 mm each, and the accuracy was 0.1 mm. The KAMOER micro-diaphragm pump EDLP600-D12B was used as the air pump, and it controlled the pump suction flow capacity by adjusting the input voltage. The National Instruments USB6289 was used as the data acquisition device. The standard gas sample contained a mixture of nitrogen and NO obtained from Harbin Liming Gas, with a NO concentration of 2050 ppm.

### 3.2. Simulation Process

(1) AIRPAK simulation process

The fan speed was set to 50 RPM, the circulating tuyere flow rate was 60 standard cubic centimeter per minute (SCCM), the Reynolds coefficient Re was 22,716.8, the Peclet number was 16,811.2, and the gas in the air chamber was turbulent. We designed two types of closed-box structures and three sampling gas pump suction flow capacity schemes. As listed in Table 1, the fan speed was set to 50 RPM.

**Figure 6:**
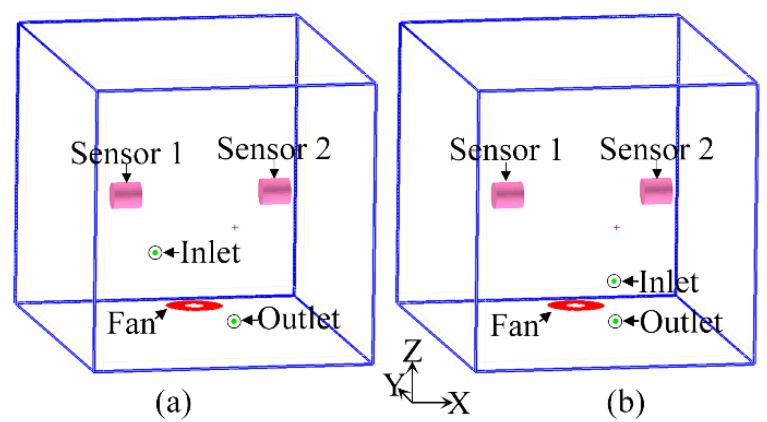
Physical models of (**a**) type I closed box and (**b**) type II closed box.

The circulating tuyere of the type I closed box was in the opposite position. The outlet port was at Y = 0 m on the X-Z plane, and the inlet port was at Y = −0.4 m on the X-Z plane. A diagram of the physical model is shown in Figure 6a. The type II closed box had a circulation outlet port on the X-Z plane and an inlet port on the X-Z plane at the position of z = 0.05 m. Figure 6b shows a diagram of the physical model.

The length (X-axis), width (Y-axis), and height (Z-axis) of the closed box are 0.3, 0.4, and 0.3 m at 25 °C, respectively. The flow rate of the air supply port is the same as that of the air return port, and, thus, it cannot bear the heat load. The air inlet temperature is the ambient temperature, and the closed box is filled with human-exhaled air. The concentration of NO is 50 ppb, and the volumes of the other gases are as follows: 78% N_2_, 16% O_2_, and 4% CO_2_. The outer wall of the electronic nose has no heat exchange with the outside world, and, thus, it is considered an adiabatic wall. The internal fan is set to flow in the opposite direction to the linear fan. For the zero pressure of the fan, the volume flow rate is 417 cm^3^/s, and the fan’s static pressure is 1300 Pa. The electronic chamber model is divided into 94,256 grids and 101,520 nodes, and the κ-ε two-equation model is used for iterative calculations.

(2) ANSYS simulation process

The inner diameter and length of the air inlet pipe of the bionic chamber were 5 and 10 mm, respectively, and those of the bionic chamber were 20 mm each. The structure of the chamber was symmetrical. The center-to-center distance between the two chambers was 24 mm. The diameter and height of the hollow cylinder at the end of the chamber were 30 mm and 3 mm, respectively. The center distance between the two sieve plates was 10 mm, and each plate had eight holes with diameters of 1 mm. Turbulence was generated in the bionic chamber by a pump suction flow capacity of 200–1200 SCCM, at which gas molecules are known to have eddy viscosity that is beneficial to the reaction between gas molecules and electrochemical sensors. We set six pump suction flow capacities: 200, 400, 600, 800, 1000, and 1200 SCCM. The shear-stress transport (SST) k-ω model was selected for calculations.

### 3.3. Experimental Data Collection

As shown in Table 2, six gas concentrations in the range of 5–200 ppb were chosen, and the sensor response and pump suction flow capacity data were collected at six pump suction flow capacities between 200 SCCM and 1200 SCCM. Prior to use, the sensor was preheated by power for 5000 s. The data acquisition frequency of the card was 10 Hz. Each set of experiments was simulated for 5 min, and after each acquisition, the electronic nose system was cleaned with dry air for 10 min before the subsequent experiment was conducted. Two experiments were conducted with the same pump suction flow capacity and gas concentration in 72 sets of experiments. One set of collected data was used for training, whereas the other set was used for testing.

## 4. Results and Discussion

### 4.1. AIRPAK Simulation Results

**Figure 7:**
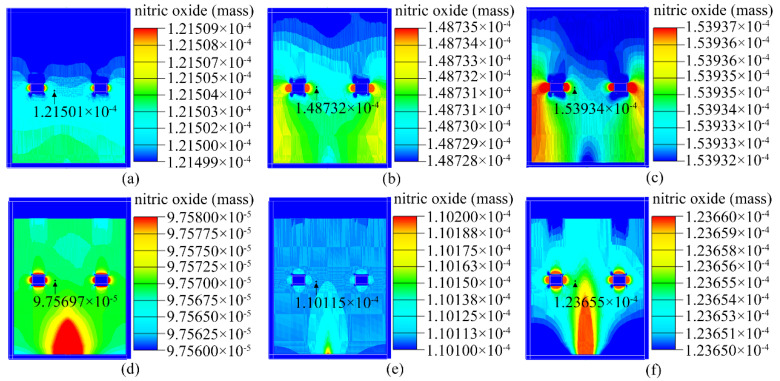
Mass of NO in the closed box. Type I electronic nose at (**a**) 200 SCCM, (**b**) 600 SCCM, and (**c**) 1000 SCCM. Type II electronic nose at (**d**) 200 SCCM, (**e**) 600 SCCM, and (**f**) 1000 SCCM.

Figure 7 depicts the results of the AIRPAK simulations. Because different models of the closed box and pump suction flow capacity were used, the NO concentration near the sensor in the closed box was also distinct.

Based on the observed results, the type I closed box was chosen for the construction of the experimental system; this is because the concentration of NO approached its actual value as the pump suction flow increased in the type I closed box.

### 4.2. System Response

**Figure 8:**
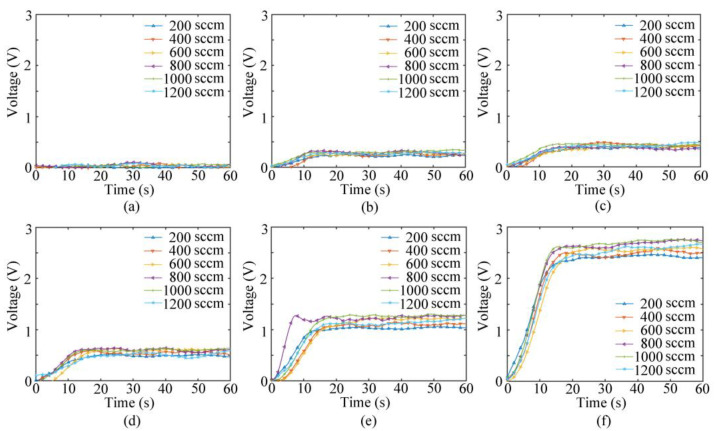
Voltage value response diagrams of bionic electronic nose system: (**a**) 5 ppb, (**b**) 25 ppb, (**c**) 35 ppb, (**d**) 50 ppb, (**e**) 100 ppb, and (**f**) 200 ppb.

Figure 8 depicts the response voltage of the experimental system to NO gas under the impact of different pump suction flow capacities using the pumping electronic nose bionic chamber. After the initial and change phases, the response curve reaches equilibrium. The impact of the pump suction flow capacity on the system is evident. The trends of the response curves for the same concentration of gas under different pump suction flow capacities between 200 and 1200 SCCM are identical. However, the magnitude of the response voltage varies.

The system response indicates that the gas collection method in the bionic chamber of the pumping electronic nose can effectively detect NO gas at ppb concentrations. The response of the electronic system depends on both the gas concentration and the pump suction flow capacity of the sampling gas. This can be further confirmed via ANOVA, as shown in Table 3. A homogeneity test of variances and a normality test are conducted, and the results are shown in Tables S1 and S2 (Supplementary Materials). The *p*-values of each observed variable indicate that the gas concentration and pump suction flow have a significant impact on the NO sensor response.

### 4.3. Comparison and Analysis of Different Pump Flows of Sampling Gas

From the original data and those obtained after the flow correction, it was observed that the impact of each concentration of the sampling gas on the response of the electronic nose system under the action of different pump suction flow capacities exhibited a certain pattern. Table 4 presents a least significant difference (LSD) comparison of the NO sensor responses at various pump suction flow capacities. It was observed that there was no significant difference between the impacts of the pump flow rates of 400, 600, and 1200 SCCM on the NO sensor response or between the effects of the pump flow rates of 800 SCCM and 1000 SCCM on the NO sensor transmission response. However, the mean NO sensor response at the pump suction flow capacities of 800 and 1000 SCCM was higher, and the value was more favorable than that at the pump suction flow capacities of 400, 600, and 1200 SCCM. The overall turbulence velocity within the bionic chamber was greater when the pump flow capacity was between 800 and 1000 SCCM. The molecular Brownian motion within the gas stream was stronger, the collision strength of the particles within the flowing gas was stronger, and the reaction with the surface of the NO electrochemical sensor occurred more easily. This observation is in good agreement with the results of previous studies [34,35]. The gas sensor response was highly dependent on the velocity of the gas that was measured flowing over the sensor surface.

**Figure 9:**
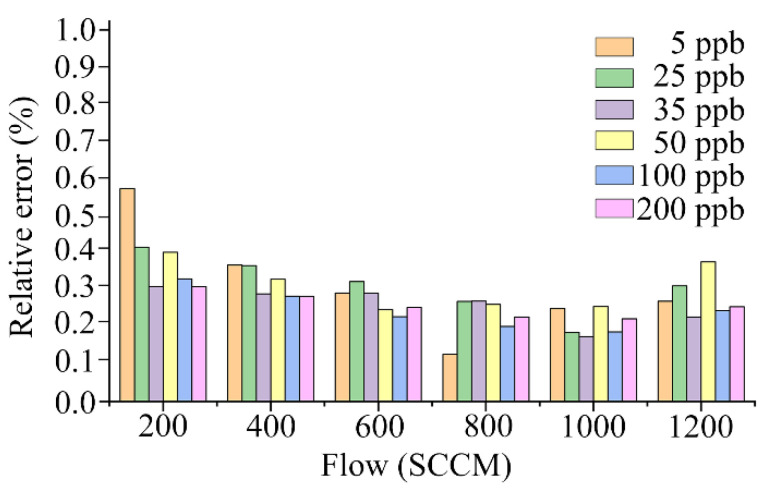
Relative errors of electronic nose system.

Figure 9 shows the relative errors of the NO sensor responses in the data obtained from the training set for the various gas concentrations and flow capacities. The mean value of the relative error of the NO sensor response was relatively high at the 200 SCCM pump suction flow capacity; similar at the 400, 600, and 1200 SCCM pump suction flow capacities; and relatively low at the 800 SCCM and 1000 SCCM pump suction flow capacities. For each concentration of the sampling gas, the relative error tended to decrease as the pump suction flow increased, and it increased with the pump suction flow after reaching 1000 SCCM. The electronic nose system that adopted the bionic chamber of the active pumping electronic nose for gas collection had the lowest relative error when the pump suction flow capacity of the sampling gas was 1000 SCCM.

**Figure 10:**
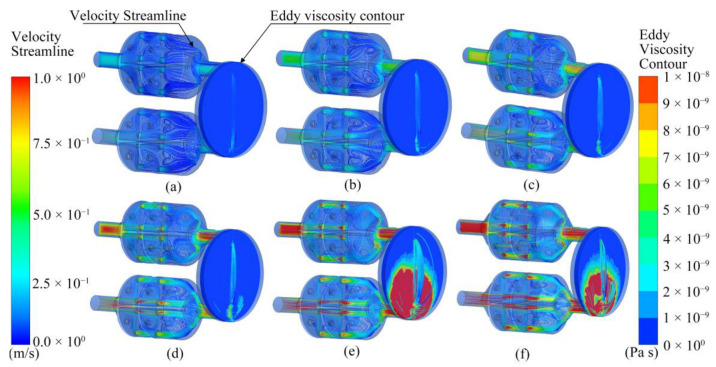
Simulation results of velocity streamline and eddy viscosity isoline in electronic nose chamber at six flow rates: (**a**) 200 SCCM, (**b**) 400 SCCM, (**c**) 600 SCCM, (**d**) 800 SCCM, (**e**) 1000 SCCM, and (**f**) 1200 SCCM.

Figure 10 shows the airflow in the ANSYS bionic chamber at different flow rates. The airflow velocity in the chamber and eddy viscosity near the sensor varied according to the pump suction flow capacity. When the pump flow capacities were 1000 SCCM and 1200 SCCM, the airflow velocity in the bionic chamber was the highest; the locations were at the sieve plate hole and at the end of the chamber. When the pump flow capacity was 1000 SCCM, the eddy current viscosity that was close to the sensor was at its peak. In this instance, the gas particles collided the most violently, exchanged momentum the quickest, and were more likely to react with the electrochemical sensor surface.

### 4.4. Correction Results and Analysis of Gas Flow Data

(1) Parameter selection and optimization based on MR and GA-BP optimization algorithm

The linear fitting and quadratic fitting calculations were adopted based on the MR algorithm. The *p*-values by the *t*-test and F-test, and the adjusted judgment coefficient R^2^, root mean square error (RMSE), Akaike’s information criterion (AIC), and other indicators were compared to obtain the quadratic fitting expression shown in Equation (15). The results of a comparative analysis of the MR fitting parameters are shown in Table S3 (Supplementary Materials).

(15) $$y=0.41225+1.3929x_{1}-0.35554x_{2}-0.063957x_{1}x_{2}+0.075741x_{2}{}^{2}$$

where y is the output corrected by the MR model, x_1_ is the output of the electronic nose sensor, and x_2_ is the gas pump suction flow.

The BP neural network model that was optimized using a GA consists of a single hidden layer with two neurons. To prevent over-fitting during machine learning, 180 data sets from the training set were randomly divided into two groups of 90 data sets each. For model training, 90 data sets were used as training samples, and 90 were used as verification samples. The weights and thresholds were optimized using a GA. Further, the number of iterations of the algorithm was set to 50, and the population size was 5.

**Figure 11:**
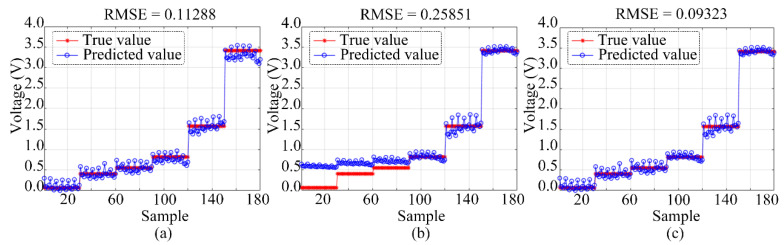
Corrected RMSE of test data set: (**a**) MR model, (**b**) GA-BP model, and (**c**) MR combined with GA-BP model.

The optimization algorithm was used to maximize the benefits of both algorithms by combining MR with GA-BP. Figure 11 demonstrates that, when the gas concentration of the MR algorithm was below 50 ppb, the impact of the system regression prediction was more accurate than when the gas concentration was above 50 ppb. However, by using the GA-BP algorithm, the difference between the system regression prediction value and the actual value decreased as the sample gas concentration increased. The system regression prediction effect was at its peak when the gas concentration was 200 ppb. Consequently, the data set may be automatically discriminated based on the MR and GA-BP optimization algorithm by using the average sensor response when the sampling gas concentration is 50 ppb, and the pump suction flow capacity for the discrimination condition is 1000 SCCM. When the response value of the NO sensor is greater than this value, the system corrects it using the GA-BP algorithm. When the response value of the NO sensor is less than this value, the system performs corrections based on the MR algorithm. The RMSE that was detected by the electronic nose monitoring system based on the optimized algorithm was reduced by 2% and 16% compared to that of the electronic nose monitoring system based on the MR algorithm and that of the electronic nose monitoring system based on the GA-BP algorithm, respectively.

(2) Optimization algorithm’s flow capacity correction results based on MR and GA-BP

To further validate the impact of the system on traffic correction, 180 sets of test data were incorporated into the traffic correction model. Table 5 displays the principal correction indices for each flow correction model. It can be seen that, after implementing the optimization algorithm based on MR and GA-BP, the original algorithm’s deficiencies were addressed, and the correction effect was significantly enhanced.

The output results of the system after the flow rate correction using the optimization algorithm are displayed in Table 6 for all sample data under a pump suction flow capacity of 1000 SCCM for different gas concentrations. After the flow correction, the precision of the active pumping bionic chamber electronic nose system based on the MR and GA-BP optimization algorithm increased from ±17.40% to ±6.86%.

Thus, when the pumping gas collection mode is used in an electronic nose system, the pump suction flow capacity has a significant impact on the sensor response. Further, the flow correction model can reduce the detection error of the system and subsequently improve the detection accuracy. This is consistent with the findings of other researchers [33,34,35,36], who believe that the gas sensor response is related to the gas flow rate. However, these studies did not integrate machine learning algorithms to correct the pump flow rate or the sensor response.

Based on a comparison of the correction accuracies of the gases with varying concentrations, as shown in Table 6, it is evident that the prediction error of the high-concentration gas using the flow correction method is less than that of the low-concentration gas. Future studies will be primarily focused on the improvement of the concentration of the gas to be measured using the enrichment method. In addition, if the system is used in a human experiment involving the detection of FeNO, the impacts of temperature and humidity differences in human-exhaled air on the system must be investigated. In this study, the gas collection in the bionic chamber of the pump electronic nose was combined with a flow correction optimization algorithm, which provides a research concept for trace-gas detection at the ppb level, thus aiding the development of household health-monitoring equipment that can take daily measurements of FeNO.

## 5. Conclusions

In this study, an active pumping bionic chamber electronic nose system was designed using system simulation modeling, and the impact of the sampling gas pump suction flow capacity on the system response was studied according to a variance analysis. An optimization algorithm based on MR and GA-BP was proposed to further improve the detection accuracy of the system. The data set could be automatically differentiated based on the concentration of the gas that was measured in order to select the appropriate flow correction model for calculations.

The NO gas exhaled by the human body was replaced with a standard gas, and the electronic nose system was used for gas collection. The detection experiments were based on six different pump suction flow capacities in the range of 200–1200 SCCM. The results indicate a nonlinear relationship between the relative error of the electronic nose system and the pump suction flow capacity. When the pump suction flow capacity was in the range of 200–1000 SCCM, the relative error of the system decreased as the pump suction flow capacity increased. However, when the pump suction flow capacity was in the range of 1000–1200 SCCM, the relative error of the system increased with the pump suction flow capacity. The pump flow capacity at the critical point was 1000 SCCM, and the relative error of the system was minimal. The detection error of an electronic nose system can be effectively reduced by utilizing an optimization algorithm based on MR and GA-BP to correct the system flow, and the system’s detection accuracy can be further enhanced. Prior to the flow correction, the accuracy of the system was 17.40%, and after the flow correction, it was 6.86%. The use of the gas collection scheme of the bionic chamber of the active pumping electronic nose in conjunction with the flow correction optimization algorithm can help to improve the detection accuracy of trace NO gas. The findings and outcomes of this study can aid in the development of household health-monitoring equipment that can take daily measurements of FeNO and can serve as a research method for trace-gas detection.

## Figures and Tables

**Table 1:** Simulation scheme.

Simulation Scheme	Closed-Box Structure	Pump Suction (SCCM)
1	type I	200
2	type I	600
3	type I	1000
4	type II	200
5	type II	600
6	type II	1000

**Table 2:** Gas concentration and pump suction flow capacity.

Serial Number	Concentration (ppb)	Flow Capacity (SCCM)	Number of Samples
1	5	200	2
2	25	400	2
3	35	600	2
4	50	800	2
5	100	1000	2
6	200	1200	2

**Table 3:** Inter-subject effect test.

Factor	Mean Square	F	*p*-Values
Concentration × Flow capacity	0.009	1.328	0.153
Concentration	26.550	4070.496	0
Flow capacity	0.078	11.964	0

**Table 4:** LSD comparison of NO sensor responses.

Flow Capacity (SCCM)	Number of Cases	Subset			
		1	2	3	4
200	30	0.7930360			
400	30		0.8395970		
1200	30		0.8688020	0.8688020	
600	30		0.8868667	0.8868667	0.8868667
800	30			0.9118807	0.9118807
1000	30				0.9344510
*p*-values		1.000	0.064	0.101	0.062

**Table 5:** Prediction indices for verification set algorithms.

Modified Model	R^2^	MSE	RMSE
MR	0.98768	0.094584	0.1241
GA-BP	0.94544	0.1956	0.26119
Optimized model	0.99113	0.075204	0.10531

**Table 6:** Precision of flow correction model.

Concentration	Pre-Correction	MR Combined with GA-BP Algorithm
5	4.585	6.513
25	21.388	26.709
35	27.468	34.115
50	39.152	50.088
100	82.179	99.072
200	158.815	200.921
Precision (±%FS)	17.40	6.86

## Data Availability

All raw experimental data used in this paper are stored at https://doi.org/10.6084/m9.figshare.21761078 accessed on 1 January 2023.

## Supplementary Materials

The following supplementary materials can be downloaded at doi.org/10.6084/m9.figshare.21776306, Table S1: Homogeneity Test of Variances; Table S2: Normality Test; Table S3: Comparative Analysis of MR Fitting Parameters.
